# Progress in Surgery

**Published:** 1894-11-24

**Authors:** 


### Progress in Surgery.

### SURGERY OF THE LIVER AND GALL BLADDER.

**Hydatid of Liver.**—R. Jones reports[1] a case of hydatid of the liver, having its centre to the left of the right every direction hypochondrium, and extending about four inches in The tumour moved up and down with respiration. An incision was made in the middle line of the abdomen, just below the ensiform cartilage. Some of the contents, which were of the usual hydatid character, were removed by aspiration. An incision about three-quarters of an inch long was then made into the cyst, and about 120 ounces of fluid escaped. The opening in the cyst was then attached to the abdominal incision. Large portions of the cyst wall were then removed by forceps, and the cavity washed out with boracic lotion. A drainage tube was inserted, and the cavity regularly washed out. Discharge continued for four or five months, and for the first six weeks portions of the cyst wall were found in the dressings. The patient left the hospital cured.

**Abscess of Liver.**—Dr. Vintras reports a case which was operated upon by the usual method, in two stages. The patient died on the ninth day. Post mortem: an abscess cavity was found at the site of puncture, which led by various sinuses into a huge abscess cavity occupying all the remainder of the right lobe, and still containing over a pint of pus. The left lobe was not affected. As regards diagnosis, Mr. Morton says[2] if there is marked bulging over the lower ribs; if the enlargement of the liver downwards is quite out of proportion to the extension of dulness upwards; if there is bulging of the anterior surface of the liver, as well as extension downwards; if we can withdraw pus at a level below the level of the pleural cavity, or just above the limit, and yet only at such a depth that we must almost certainly have gone through the pleural cavity (which here is only a very narrow space, even when full of fluid) into the liver beyond; if an extension of hepatic dulness upwards moves distinctly downwards with respiration; and, lastly, if our patient has been in a tropical climate, and has presented latent symptoms of obscure illness with a rigor at the commencement, and perhaps repeated, then we may suspect, or in the presence of some of these conditions actually diagnose, hepatic abscess. The differential diagnosis of sub-diaphragmatic abscess on the right side from hepatic abscess can scarcely be made before exploration.

M. Zancarol,[3] allowing for the inevitable mortality from multiple liver abscess, estimates the mortality in his own practice in single liver abscess operations as follows: 120 cases operated on by trocar, mortality 72 per cent., post-operation fistulæ, 19 per cent.; 41 cases operated on by knife, drainage and antiseptics, mortality 62 per cent., 17 per cent. post-operation fistulæ; 157 cases operated on by the method to be described, mortality 29 per cent., no post-operation fistulæ. The operation from which M. Zancarol obtained the favourable results last mentioned is as follows: For abscess of the left lobe, in which there is little risk of serious hæmorrhage, he uses the knife; in operating on the right side, where the risk of hæmorrhage is considerable, he uses the thermo-cautery. In the latter case, after ascertaining by means of the aspirator needle, the situation of the abscess, he divides the integuments at a point corresponding to what he supposes to be the bottom of the upper third of the abscess. The line of incision is made parallel to the ribs, and to an extent corresponding to the assumed breadth of the abscess. A portion of the rib is then excised to the full length of the incision, and the surface of the liver exposed. The abscess is opened by a small incision, the needle of the aspirator being used as a guide. A finger passed into the cavity plugs the wound, hooks the liver against the chest wall, and serves as a guide for the two retractors, which are then inserted and committed to an assistant, who keeps the liver close to the chest wall, so as to prevent escape of pus into the serous cavities. The abscess is now emptied, and the incision in the liver extended so as to correspond to the incision in the skin. The cavity is flushed with a solution of salicylic acid (1 to 1,000); then the walls are wiped clean with mounted sponges or cotton. This cleansing is carried so far as to remove all sloughs and broken-down tissue, and expose sound tissue, anything like scraping or violence being avoided. The cavity is stuffed with iodoform gauze, and an antiseptic dressing applied. The second dressing is generally made on the third or fourth day, or earlier, according to indications.

**Wound of the Liver.**—T. Gann reports[4] a case in which the spear of a harpoon penetrated the liver from immediately below the ensiform cartilage to the posterior border of the right lobe. Hæmorrhage was rather free after the removal of the harpoon. The abdominal wound was sutured, and a drainage tube left in the track of the harpoon for five days. The patient was discharged, with the wound healed, on the twenty-first day.

[1] Lancet, April 7th, 1894, p. 860. [2] Brit. Med. Journ., Mar. 31st, 1894, p. 682. [3] Lancet, May 12th, 1894, p. 1181. [4] Lancet, June 2nd, 1894, p. 1371.

